# Supplementary material for: Energy efficiency and biological interactions define the core microbiome of deep oligotrophic groundwater
Source: Nat Commun. 2021 Jul 12;12:4253. doi: 10.1038/s41467-021-24549-z (PMC8275790; doi:10.1038/s41467-021-24549-z)
Supplement: Supplementary file 1 — Supplementary information [file 41467_2021_24549_MOESM1_ESM.pdf]

Supplementary Information

Energy efficiency and biological interactions define the core microbiome of deep oligotrophic groundwater

Mehrshad, et al.

Supplementary Figures and legends

Supplementary Background

Supplementary Methods

Supplementary Figures and legends

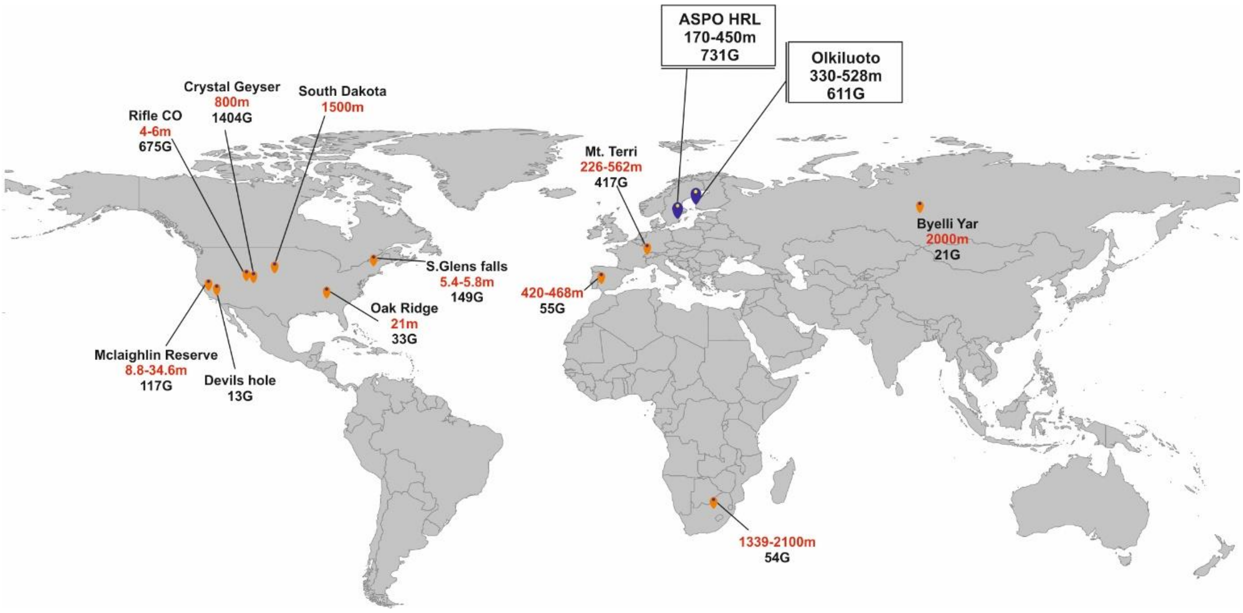

Supplementary Fig. S1- Geographical distribution of publicly available metagenomic datasets sequenced from oligotrophic groundwater samples (landfill groundwater, oil-influenced, and shale samples are not included in this representation). The depth ranges (as mentioned in the corresponding publication) of the samples (in red) and the amount of publicly available sequenced data (presented as Gb) are shown for each location.

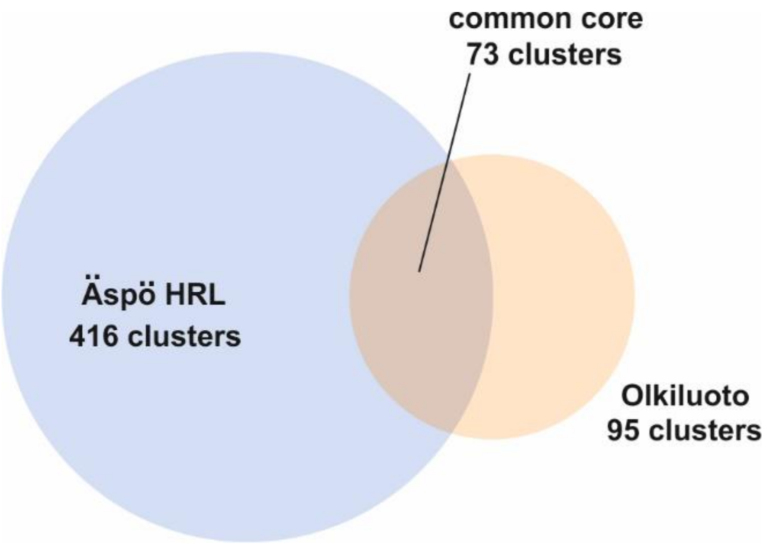

Supplementary Fig. S2- Overlap of the microbiome of the Fennoscandian Shield deep groundwater flowing in two disconnected sites. Site name and number of clusters are mentioned in the diagram.

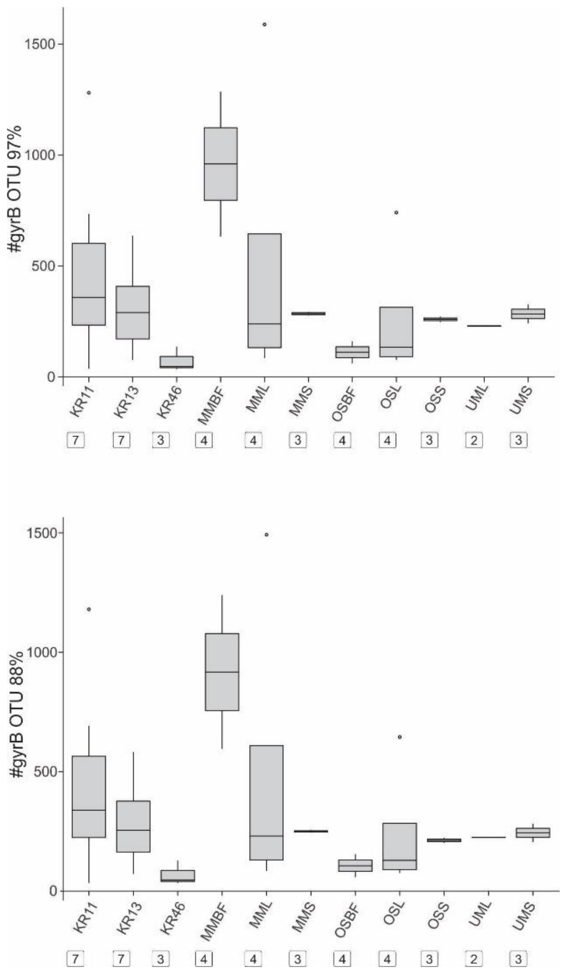

Supplementary Fig. S3- Species richness in metagenomes generated from each deep groundwater site based on gene gyrB (centerline, median; hinge limits, 25 and 75% quartiles; whiskers, 1.5x interquartile range; points, outliers). Numbers next to the sample names represent the number of metagenomes generated and analysed for this figure.

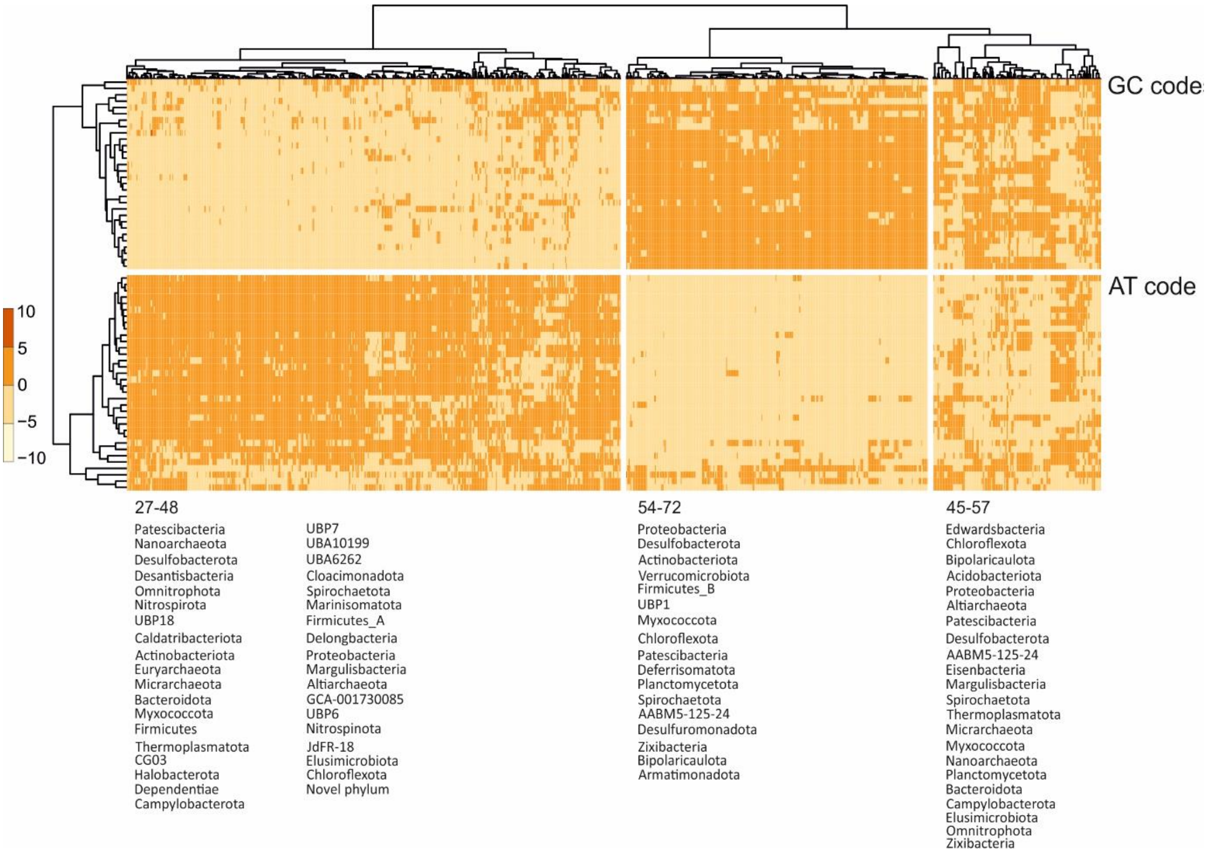

Supplementary Fig. S4- Representation of the frequency (the expected number of codons, given the input sequences, per 1000 bases) of utilization of synonymous codons across MAGs and SAGs of the FSGD.

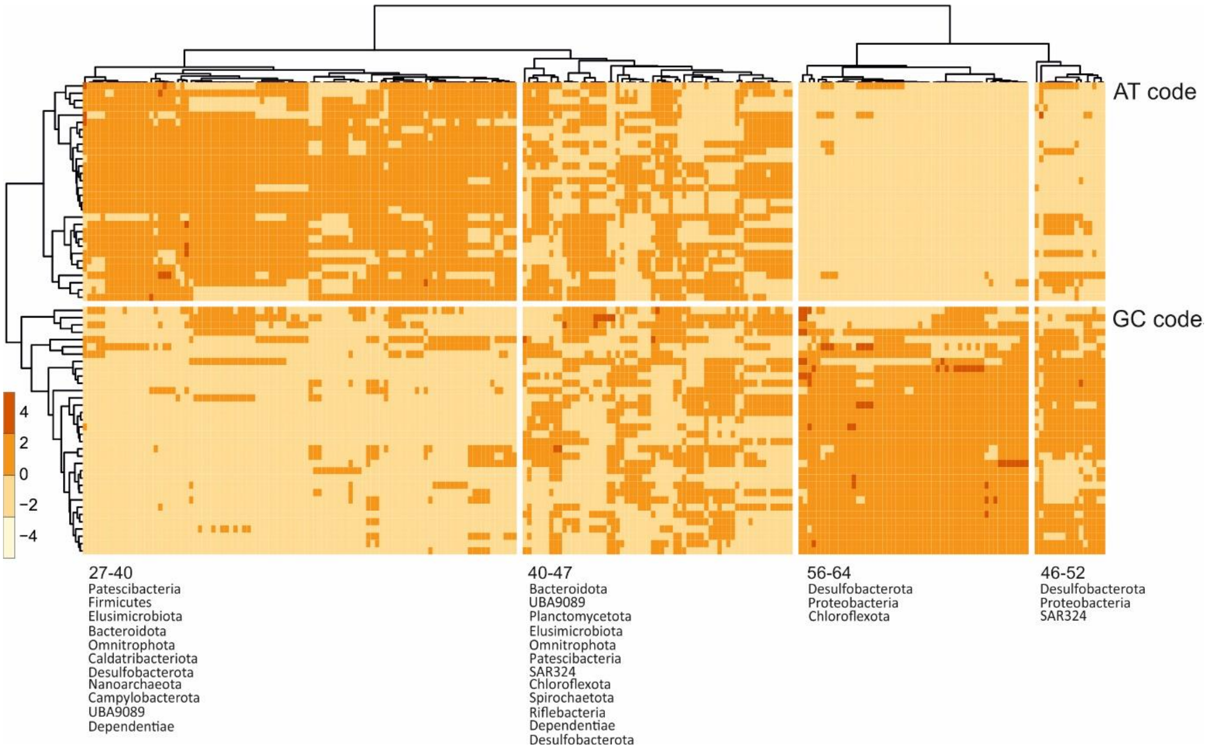

Supplementary Fig. S5- Representation of the frequency (the expected number of codons, given the input sequences, per 1000 bases) of utilization of synonymous codons across highly expressed (TPM >10000 arbitrary threshold) MAGs and SAGs of the FSGD.

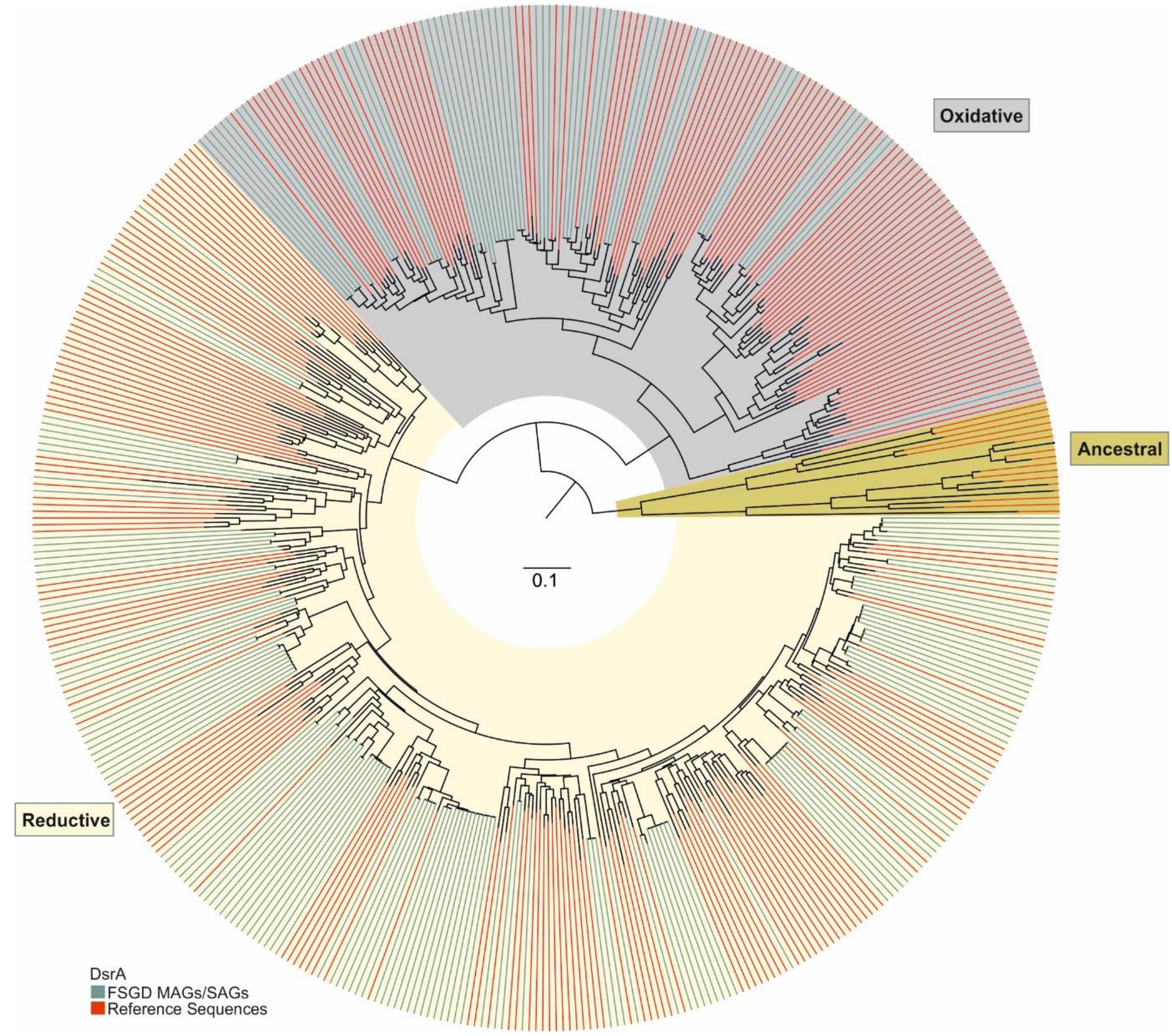

Supplementary Fig. S6- Reconstructed phylogeny of the DsrA protein sequences recovered from FSGD reconstructed MAGs and SAGs (dark blue) together with reference DsrA sequences (in red). Different types of DsrA protein according to their function are mentioned in the figure. The alignment and the phylogenetic tree are publicly available via figshare at DOI:10.6084/m9.figshare.14166638 and DOI:10.6084/m9.figshare.14166650.v1 respectively.

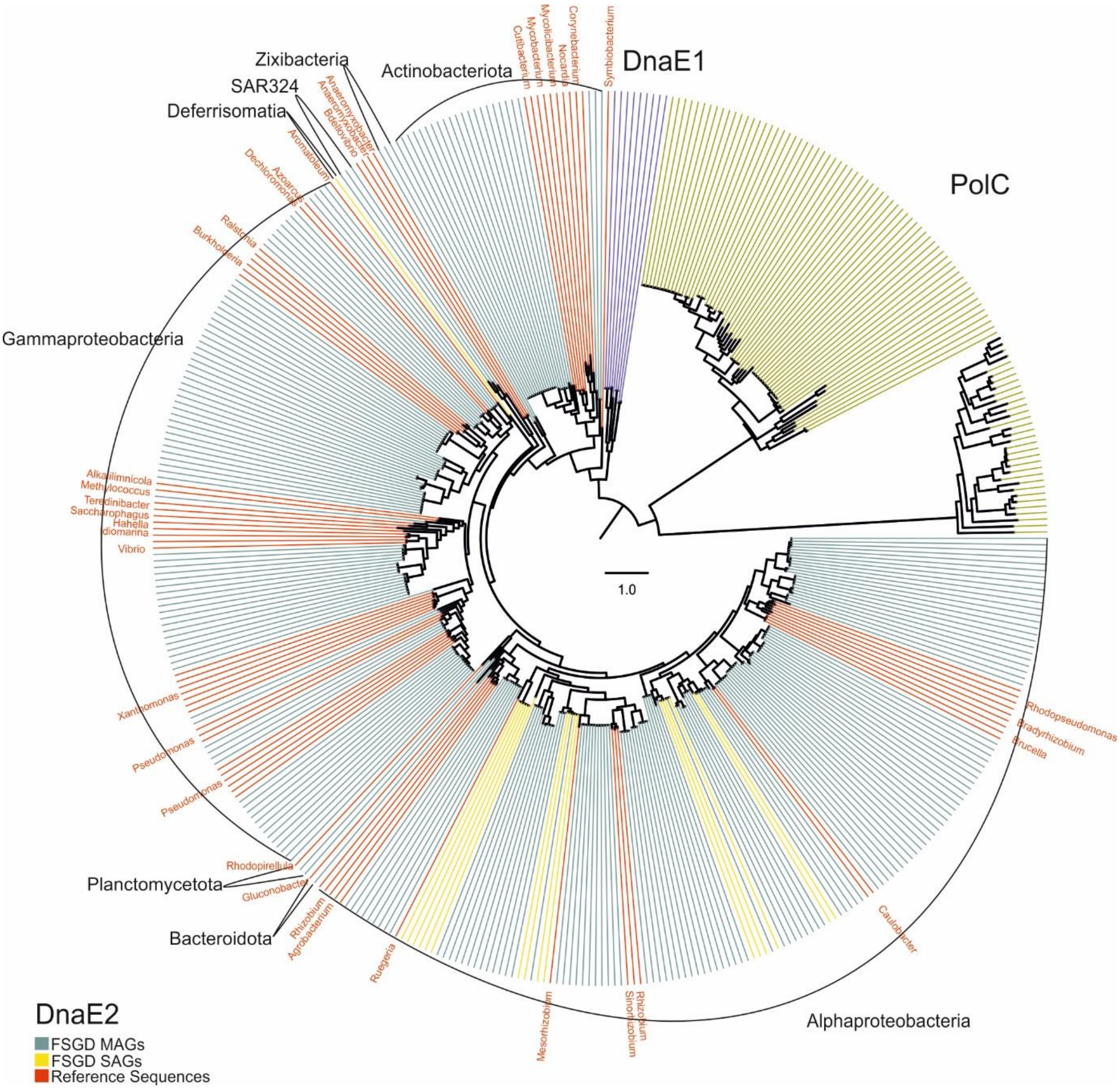

Supplementary Fig. S7- Reconstructed phylogeny of the C-family polymerases. The tree is arbitrarily rooted by the PolC branch. Taxonomy of the leaves are written for each group. The reference genomes are the reviewed sequences retrieved from uniprot for each polymerase type (red). The alignment used to reconstruct this phylogeny and the unrooted tree are available via figshare at DOI:10.6084/m9.figshare.12170310.v1 and DOI:10.6084/m9.figshare.13298513 respectively.

Background

During the last decades, it has been demonstrated that microorganisms inhabit the sub-surface biome down to several kilometers below the surface[1]. Although microbial life in the deep crust biosphere has been gaining interest[2], due to its difficulty to access it is still one of the least understood environments on earth[3]. Consequently, many novel taxa are being identified in the deep biosphere[4,5].

The continental deep biosphere is estimated to contain 2 to 6 × 10^{29} cells[6,7], containing members from all domains of life along with viruses[8–14]. Even though the available energy flux is very low compared to that of the photosynthetically fixed carbon on the surface[15], microorganisms are widely spread in oceanic crust fluids[16–18], marine sediments[19,20], terrestrial rocks[21,22], and granitic groundwaters[23–25]. In addition, many of these deep biosphere microbes are both alive and active[10,26–30].

In continental groundwaters, microbial activity is strongly positively correlated to the proximity of the photosynthesis-fueled surface[7] and thus, water-bearing deep fracture systems are extremely oligotrophic[31]. A recent study showed that there is a steady flow of surface organisms to the deep subsurface with a selection event resulting in some taxa adapting to the new conditions while others perish[24]. In addition, Lopez-Fernandez et al.[28] showed the presence of viable taxa in a deep continental crystalline rock and that any non-viable cells are rapidly degraded and recycled into new biomass[28]. The deep biosphere is suggested to be adapted to the low energy conditions by e.g. small cell size and streamlined genomes[32]. Microbial populations with the potential to initiate biofilm formation were also identified in deep terrestrial subsurface waters[11]. This close proximity likely promotes syntrophy and cycling of nutrients between populations[33].

Site lithologies

The two sampling locations, Äspö HRL and Olkiluoto Island drillholes, are situated on opposite sides of the Baltic Sea on the Swedish (Lat N 57° 26′ 4′′ Lon E 16° 39′ 36′′) and Finnish (Lat N 61° 14′ 31′′, Lon E 21° 29′ 23′′) coasts. The crystalline bedrock of Sweden and Finland is part of the Precambrian Fennoscandian Shield that is predominantly made up of granite and quartz monzodiorite of quartz and aluminosilicate minerals including mica and feldspar.

The bedrock in the Äspö HRL region consists of overall well preserved (locally low-grade metamorphism and discrete foliation occur) Palaeoproterozoic ~1.8 Ga granitoids of the Transscandinavian Igneous Belt[34]. At the Äspö HRL site, these rocks have a composition ranging from diorite to granite[35]. The rocks are also cut by a number of deformation zones and open fractures, which frequently have surfaces covered by high and/or low temperature 1-15 mm thick secondary-mineral precipitates including calcite, chlorite, pyrite, clay minerals, epidote, adularia, and hematite[36]. Hence, the fluids flowing in the fractures are in direct contact with both Precambrian granitoids and a variety of secondary minerals of variable age.

Olkiluoto is located in the southern Satakunta region in south-western Finland. The ~1.8 Ga Palaeoproterozoic bedrock of the southern Satakunta region is composed of supracrustal, metasedimentary and metavolcanics rocks deformed and metamorphosed during the Svecofennian orogeny[37]. The main rock types at Olkiluoto are mica and veined gneisses, migmatite granite, grey gneisses and diabase. Minor veins and dykes are quartz feldspar gneisses and amphibolites. Mica and veined gneisses contain calcite, sulfphides and clay mineral fracture fillings[38].

Supplementary Methods

In this study, the extremely oligotrophic deep groundwaters of two sites excavated in the Fennoscandian Shield have been sampled and extensively analyzed via a “multi-omics” approach.

Site description

The Äspö Hard Rock Laboratory (HRL) is located in the south-east of Sweden (Lat N 57° 26′ 4′′ Lon E 16° 39′ 36′′) and is excavated in the Proterozoic crystalline bedrock of the Fennoscandian Shield extending to a depth of 460 m below sea level (mbsl) with 3600 m of total tunnel length[42]. The tunnel provides access to investigate the microbial life in the deep Fennoscandian Shield groundwater.

Building of the Äspö Hard Rock Laboratory was initiated in 1986 and the site circumvents many problems normally associated with sources of contamination in groundwater. While flow of groundwater towards the tunnel results in mixing of some groundwaters, it also flushes away any anthropogenic contamination introduced into the deep biosphere. In addition, boreholes drilled far into the bedrock means that oxygen does not penetrate to the sampled fracture waters and enable waters to be sampled under *in situ* conditions. Finally, the risk of influencing the microbial community due to materials used to close the borehole sections[43] is minimized by flushing three section volumes prior to sampling.

The island of Olkiluoto on the south-west coast of Finland will host a deep geological repository for the final disposal of spent nuclear fuel (Lat N 61° 14′ 31′′, Lon E 21° 29′ 23′′). Groundwater is accessed via deep drillholes at Olkiluoto fitted with multipackers that allow isolation of fracture fluids. Fractures were pumped for 2–3 months prior to microbiological

sampling. During this time, chemical parameters (including dissolved O_2 and oxidation-reduction potential) were monitored to ensure that the water was representative of the isolated fracture. Deep drillholes and excavation of the underground tunnels at Olkiluoto can form transient drawdown of groundwater in connected fractures due to changes in hydraulic head (pressure). This can also cause mixing of different groundwater-types at Olkiluoto, but deep groundwaters are highly reducing and there is no evidence of oxygen penetration[44]. Sterility and anoxic conditions during sampling of groundwaters at Olkiluoto, Finland, were ensured by filtering directly through 0.2 µm filters as described in[33].

Groundwater samples

We collected groundwater samples from five different boreholes: SA1229A-1 (171.3 mbsl), KA3105A-4 (415.2 mbsl), KA2198A (294.1 mbsl), KA3385A-1 (448.4 mbsl), and KF0069A01 (454.8 mbsl) with varying geochemical conditions as described below. A total of 27 metagenomes were generated from the respective Äspö HRL boreholes that are listed in Supplementary Data 1. The biofilm metagenomes were formed on rock and glass surfaces after 33 days in flow cells attached to the KA2198A and KF0069A01 boreholes (total $n = 8$) as described in Wu et al[11]. Further metagenomes were generated from planktonic cells captured on 0.22 µm filters from boreholes SA1229A, KA3105-4, and KA3385A (total $n = 6$; termed 'large cells') and cells that passed through the 0.22 µm filters from same three boreholes ($n = 9$; termed 'small cells') as previously described[32]. Finally, planktonic cells captured on 0.1 µm filters from boreholes SA1229A and KA3385A that were extracted in this study using the MOBIO PowerWater DNA Isolation Kit or phenol-chloroform ($n = 4$).

These groundwaters carried iron as Fe^{2+}, contain dissolved sulfide (HS^-), had temporally stable chemistry and $\delta^{18}O$, and neutral pH[32]. However, differential chemical composition and $\delta^{18}O$ values enable us to characterize the origin and age of these groundwaters[45]. Groundwaters

SA1229A-1, KA3105A-4, and KA2198A have stable chloride concentrations and $\delta^{18}O$ values, similar to those corresponding to Baltic Sea water[46]. Consequently, these groundwaters have a marine signature and are most likely composed of Baltic Sea water mixed with minor proportions of meteoric water and/or older more saline water residing in the bedrock fractures[47]. The precise infiltration age of this groundwater was unknown, but estimated to be <20 years or even less[11]. They were termed 'modern marine' ('MM') waters, concretely 'MM-171.3' for SA1229A-1, 'MM-294.1' for KA2198A, and 'MM-415.2' for KA3105A-4 groundwaters. Borehole KA3385A-1 contained water with chloride concentrations and $\delta^{18}O$ values in between the groundwaters with saline signature with a very long residence time[48] and marine groundwaters. Therefore, this groundwater was classified as thoroughly mixed ('TM-448.4') and is composed of unknown proportions of two or more water types such that the age of this groundwater cannot be assessed[25]. Borehole KF0069A01 had a typical signature of low dissolved organic carbon and other anions, high chloride and sulfate concentrations derived from mineral weathering, an age of millions of years[11], and was defined as 'old saline' ('OS-454.8'). The MM-171.3, MM-415.2, and TM-448.4 boreholes were sampled from November to December 2016 while MM-294.1 and OS-454.8 were both sampled between May to June 2013 (Supplementary Data 1).

Olkiluoto, Finland. Groundwater was collected from three drillholes that access fracture fluids at different depths; OL-KR11 (366.7-383.5 mbsl), OL-KR13 (330.5-337.9 mbsl), and OL-KR46 (528.7-531.5 mbsl). Multiple samples were collected during 2016 (OL-KR11 n = 7, OL-KR13 n = 7 and OL-KR46 n = 3). Geochemical parameters of the groundwater were monitored throughout the sampling period and they are available in Supplementary Data 1. At Olkiluoto, the groundwater chemistry is stratified with depth. Salinity increases with depth and brackish sulfate-rich groundwater is found up to ~400 m depth, beyond which sulfate-free saline

groundwater dominates (Posiva 2013; http://www.posiva.fi/en/databank/posiva_reports/olkiluoto_site_description_2011.1871.xhtml#.XnkY5C2ZNTY). OL-KR11 and OL-KR13 drillholes both access brackish groundwater types (residence time 2,500-8,500 years). Drillhole OL-KR46 accesses a deeper saline groundwater with a residence time >10,000 years[44].

Biomass collection, DNA extraction, and metagenome sequencing

Äspö HRL. Planktonic cells were collected after flushing five borehole section volumes on sterile polyvinylidene fluoride (PVDF), hydrophilic, 0.1 µm, 47 mm Durapore membrane filters (Merck Millipore) under *in situ* conditions by connecting a High-Pressure Stainless Steel Filter Holder (Millipore) with a downstream needle valve and pressure gauge directly to the borehole. After filtering an appropriate volume of groundwater, each filter was rolled and placed in a sterile cryogenic tube (Thermo Scientific) and immediately frozen in liquid nitrogen. Samples were frozen at the sampling site to allow transport to the laboratory without any changes in the microbial community. Tubes were stored at -80 °C until further processing. DNA of samples MM-171.3-PC and TM-448.4-PC were extracted using the phenol/chloroform/isoamyl alcohol (24:24:1) method using Phase Lock tubes (Eppendorf). Firstly, 840 µL of TE buffer, pH 8 plus 94 µL of lysozyme (100 mg/mL) were added to each filter before incubation at 37 °C for 30 min. Then, 60 µL of 10 % sodium dodecyl sulfate (SDS) and 6 µL of proteinase K (20 mg/ml) were added, mixed, and incubated at 50 °C for 20 min. Afterwards, an equal volume of phenol/chloroform/isoamyl alcohol was added to the cell lysate, mixed by inverting, and transferred to a Phase Lock Gel tube before centrifugation at 1500 × g for 10 min. Then, another equal volume of phenol/chloroform was added and mixed before centrifuging at 1500 × g for 10 min. The nucleic acid was precipitated by adding an

equal volume of ice-cold isopropanol and 0.1 volume of 3M sodium acetate, pH 5.2 and incubating at -20 °C for 60 min. After precipitation, the nucleic acids were centrifuged at 16000 × g and 4 °C for 20 min. The supernatant was discarded, and the pellet was rinsed with 500 µL of cold 80 % ethanol. Finally, the pellet was dried at 55 °C on a heat block and re-suspended in 50 µL of TE buffer prior incubation overnight at 4°C. The next day the DNA was incubated at 70 °C for 10 min to help dissolve the last of the DNA. DNA samples termed MM-171.3-PW, MM-415.2-PW, and TM-448.4-PW were extracted using the MO BIO PowerWater DNA isolation kit, following the manufacturer's instructions except that the final DNA re-suspension was performed using 60 µL of eluent[32]. The quality and quantity of the extracted DNA by both methods were analyzed with a Thermo Scientific Nanodrop 2000 and Qubit 2.0 Fluorometer (Life Technologies), respectively. Extracted DNA was stored at -20 °C. Twenty-seven metagenomic datasets were generated from the samples collected from the Äspö HRL. Detailed statistics of the generated metagenomes and the respective sequencing platform are shown in Supplementary Data 1. DNA was extracted from the MM-294.1 and OS-454.8 samples as explained in the reference[11].

Olkiluoto. To collect biomass for DNA analysis, approximately 10 L of groundwater was pumped directly into a chilled sterile Nalgene filtration unit fitted with a 0.22 µm pore size Isopore polycarbonate membrane (Millipore) and connected to a vacuum pump. After filtration, the membrane filters were rolled and stored in 1.5 mL sterile screwcap tubes. Filters collected for DNA extraction were preserved in 750 mL LifeGuard Soil Preservation Solution (MoBio, Carlsbad, CA, United States) and transferred to the laboratory on dry ice. Filters were stored at -20 °C until further processing. DNA content was extracted using a phenol-chloroform protocol[29] with the following modifications. Firstly, filter pieces were subject to bead-beating (2 × 15 s) prior to incubation in lysozyme for 2 h at 37 °C and secondly, lysate

was incubated in Proteinase K (200 mg/mL final concentration) for 2 h. Extracted DNA was measured using the Qubit dsDNA High Sensitivity Assay kit (Thermo Fisher Scientific, Inc.). A total of 17 metagenomic datasets were generated from Olkiluoto, the statistics are shown in Supplementary Data 1.

RNA extraction and metatranscriptome sequencing

The groundwaters were sampled from the Äspö HRL under *in situ* conditions using two different sampling methods. Firstly, by connecting a sampling device with an in-built fixation system as described in Lopez-Fernandez et al.[10] from June 2015 to March 2016 (Supplementary Data 1). Secondly, by connecting a high-pressure stainless steel filter holder (Merck Millipore, USA) with a downstream needle valve and pressure gauge as described in Lopez-Fernandez et al.[25] from September 2015 to January 2016 (Supplementary Data 1). In both cases, planktonic cells were collected on sterile hydrophilic polyvinylidene fluoride (PVDF) membranes with 0.1 µm poresize (47 mm Durapore, Merck Millipore, USA) under *in situ* conditions. The cell collection using both sampling methods, RNA extraction, and cDNA generation was performed as previously described[10]. Detailed statistics of the nine sequenced metatranscriptomes are shown in the Supplementary Data 1.

Single cell collection and amplification

The metagenomics samples were augmented with 564 single-cell amplified genomes (SAGs). The SAGs originate from MM-171.3 (borehole SA1229A-1, *n*=118), MM-415.2 (borehole KA3105A-4, *n*=15), and TM-448.4 (borehole KA3385A-1, *n*=148) borehole samples from the Äspö HRL along with OL-KR11 (*n*=138), OL-KR13 (*n*=117), and OL-KR46 (*n*=28) borehole

samples from the Olkiluoto. SAGs were amplified, sequenced, and assembled by the Joint Genome Institute (JGI), USA.

SAGs were de-replicated separately from the MAGs. SAGs that are containing the exact match of 16S rRNA and those with average nucleotide identity higher than 95% were combined in order to retrieve a higher number of good quality SAGs. These combined SAGs are referred to as several-SAG (s-SAG). A total of 22 SAGs were also sequenced from different water types of Äspö HRL at the SciLifeLab, Sweden as a pilot study. The SAGs were sequenced using the Illumina platform and assembled using MEGAHIT[49].

SAGs were clustered separately from the MAGs using fastANI (v. 1.1) with 95% average nucleotide identity and 70% minimum overlap. Then, SAGs belonging to the same cluster were analyzed using the 'merge' command in checkm (v. 1.0.7) to find sets of within-cluster SAGs that could potentially be merged in order to increase the completeness. Initially, this resulted in nine pairs of SAGs where the estimated combined genome completeness would increase. GC-profiles were calculated for these 18 SAGs using 1 kbp sliding windows and similar profiles for each pair were validated by manual inspection. Next, redundancies within each pair were investigated by aligning contigs from SAGs with nucmer (v. 3.23) with default settings. Aligned regions were only kept on the longer contigs, by clipping the corresponding stretch from the shorter contigs. If clipping resulted in a contigs <300 bp, that contig was removed completely. In addition, if more than 25% of a contig aligned to another contig, the shorter contig was removed completely. After removing redundant regions, contigs from the SAG pairs were combined. All s-SAGs were again checked for completeness and contamination using Checkm and those with >5% contamination was kept as original SAGs. Detailed information regarding the SAGs is shown in Supplementary Data 1.

Species richness using gene *gyrB*

To evaluate the species richness of each ecosystem captured by the metagenomes analyzed in this study, we extracted the *gyrB* genes from all metagenomic assemblies, evaluated the annotation by checking the conserved domains of the gene, and then clustered them at the 97% and 88% identity threshold defined for this gene to reconstruct *gyrB* mOTUs [39,40] using CD-hit[41] (Supplementary Fig. S3).

Fennoscandian Shield genomic database (FSGD)

The generated "multi-omics" data were used to construct a comprehensive genomic and metatranscriptomic database of different water-types of the extremely oligotrophic deep groundwater.

The sequenced metagenomes were quality checked and trimmed using Trimmomatic[50] (v. 0.36) with settings to trim the Illumina TruSeq adapter ('TruSeq3-PE-2.fa:2:30:15 LEADING:3 TRAILING:3 SLIDINGWINDOW:4:15 MINLEN:31'). For the six samples sequenced on the MiSeq platform, reads were first cropped to 125 bp by trimming the right end of the reads prior to adapter and quality trimming as above. This was done to recover more paired-end reads from these samples that all had lower quality bases at the end of reverse reads. Each dataset was assembled separately as well as co-assemblies on those datasets originating from the same water type in each sampling site using MEGAHIT[49] (v. 1.1) with settings (--k-min 21 --k-max 141 --k-step 12 --min-count 2). Contigs ≥2kb in each assembly were automatically binned using metabat2[51] with default setting. CheckM[52] was used to estimate the genome completeness of MAGs, SAGs, and s-SAGs. Those with completeness ≥50% and contamination ≤5% were considered for down-stream analysis. In addition, SAGs with <50% completeness

were considered for down-stream analysis if they clustered with another SAG in the SAG-specific fastANI step (see above) and if they had a genome size ≥500 kbp.

Genome de-replication. MAGs and SAGs were clustered using fastANI[53] (v. 1.1) at ≥95% identity and ≥70% coverage threshold. Those genomes in a single cluster are considered as representatives of a single population.

Genome taxonomy and phylogeny. Taxonomic affiliation of the FSGD MAGs/SAGs was assigned using GTDB-tk (v. 0.2.2) with reference to the release 86 database[54]. The alignments generated by the GTDB-tk for bacteria and archaea were curated and used for phylogeny reconstruction using FastTree[55] (v. 2.1.10) with parameters '-wag -gamma'.

Gene annotation and functional analysis. Prodigal[56] (v. 2.6.2) was run in metagenomic mode ('-p meta') for predicting protein-coding genes in the assembled contigs. This was followed by functional annotation of the predicted proteins using eggnog-mapper[57] (v. 2.2.1) with the eggnog_5.0 database, and pfam_scan.pl (v. 1.6) with the 31.0 release of the PFAM database. Reconstructed MAGs and SAGs were initially annotated using Prokka[58] (v. 1.12) followed by further annotation with eggnog-mapper and pfam_scan.pl using the same databases as for the metagenomic assemblies. Enzyme EC numbers, and KEGG orthologs, pathways and modules were assigned from the eggnog-mapper output. All annotations of key genes were manually inspected for their conserved domains and their annotations were further evaluated using NLM's Conserved Domain Database (CDD) search[59] and phylogeny.

Genome presence/absence patterns. Metagenomics reads were mapped against all MAGs/SAGs that passed the criteria for downstream analysis using bowtie2[60] (v. 2.3.3.1) with parameters '--very-sensitive --no-unal'. This was followed by removal of duplicates using MarkDuplicates from the picard suite (v. 2.18.6). Only contigs that ≥50% of their length was covered by the mapped reads were considered for further analysis. Mapped reads were

counted using featureCounts[61] (v. 1.6.1) with settings '-M -B' to count multi-mapping and only count read-pairs with both ends aligned. The raw counts were normalized as transcripts per million (TPM) in order to calculate MAGs/SAGs abundance in each metagenome. Based on the calculated average TPM per contig; the MAGs/SAGs were considered detected in the metagenome if they show value ≥1 and not detected if the value is <1. These strict mapping thresholds identify closely related isolates to the reconstructed MAGs/SAGs.

Computation of Isoelectric point and codon usage frequency. The isoelectric point calculation for the protein sequences as well as amino acid features were calculated using pepstat software in the EMBOSS package (v. 6.6.0)[62]. The codon usage frequency of the coding regions was calculates using software cusp in the EMBOSS package (v. 6.6.0)[62].

DnaE2 phylogeny. The phylogeny of the C-family polymerases was reconstructed by using the reference genomes are the reviewed sequences retrieved from uniprot for each polymerase type. The annotation of the protein coding sequences with evaluated annotation as DnaE2 was verified by using this phylogeny. Sequences were aligned using Kalign[63] (2.04) and FastTree (v. 2.1.10) was used for creating the maximum-likelihood tree (JTT +CAT model, gamma approximation).

Dissimilatory sulfur metabolism. The phylogeny of DsrA as a key gene in the dissimilatory sulfur metabolism was generated by using reference *dsrA* genes (both oxidative and reductive types) together with the genes annotated as *dsrA* in the reconstructed MAGs/SAGs of our study (≥200 amino acids length). Sequences were aligned using Muscle[64] in MEGA7[65] and evolutionary relationships were visualized by constructing a maximum-likelihood phylogenetic tree (JTT +CAT model). All residues were used, and the tree was bootstrapped with 100 replicates. MAGs and SAGs containing dsrA gene were further inspected for the presence of aprAB (K00394 and K00395), *sat* (K00958), *dsrAB* (K11180 and K11181), *dsrC*

(K11179), dsrD (PF08679), and *dsrEFH* (K07235, K07236, and K07237). These genes were searched for using eggnog-mapper[57] (v. 2.2.1 with the eggnog_5.0 database) and pfam_scan.pl (v. 1.6 with the 31.0 release of the PFAM database) and annotations were manually validated for each gene. The contribution of each MAG/SAG to the sulfur cycle was inferred according to the pattern of presence/absence of these genes as suggested by Anantharaman et. al[66]. For the final inference members of each cluster were considered together.

Metatranscriptome analysis. The sequenced metatranscriptomes were quality checked and trimmed using Trimmomatic (v. 0.36) [50]. The rRNA reads were filtered out using cmsearch (v. 1.1.3)[67]. The remaining reads were mapped against the FSGD MAGs/SAGs. The expressed genetic content of each MAG/SAG were extracted at the threshold of 100 TPM and the pattern of gene expression and the expressed content of each MAG/SAG was analyzed.

References

1. Magnabosco, C. *et al.* The biomass and biodiversity of the continental subsurface. *Nat. Geosci.* **11**, 707–717 (2018).
2. Colman, D. R., Poudel, S., Stamps, B. W., Boyd, E. S. & Spear, J. R. The deep, hot biosphere: Twenty-five years of retrospection. *Proc. Natl. Acad. Sci.* **114**, 6895–6903 (2017).
3. Cario, A., Oliver, G. C. & Rogers, K. L. Exploring the Deep Marine Biosphere: Challenges, Innovations, and Opportunities. *Front. Earth Sci.* **7**, 1–9 (2019).
4. Sackett, J. D. *et al.* Four draft Single-Cell genome sequences of novel , nearly identical Kiritimatiellaeota strains isolated from the continental deep subsurface. *Microbiol Resour Announc* e01249-18 (2019).

5. Anantharaman, K. *et al.* Thousands of microbial genomes shed light on interconnected biogeochemical processes in an aquifer system. *Nat. Commun.* **7**, 1–11 (2016).
6. Flemming, H.-C. & Wuertz, S. Bacteria and archaea on Earth and their abundance in biofilms. *Nat. Rev. Microbiol.* **17**, 247–260 (2019).
7. Magnabosco, C. *et al.* A metagenomic window into carbon metabolism at 3 km depth in Precambrian continental crust. *ISME J.* **10**, 730–741 (2016).
8. Al-Shayeb, B. *et al.* Clades of huge phages from across Earth's ecosystems. *Nature* (2020) doi:10.1038/s41586-020-2007-4.
9. Schwank, K. *et al.* An archaeal symbiont-host association from the deep terrestrial subsurface. *ISME J.* **13**, 2135–2139 (2019).
10. Lopez-Fernandez, M. *et al.* Metatranscriptomes reveal that all three domains of life are active but are dominated by Bacteria in the Fennoscandian crystalline granitic continental deep biosphere. *MBio* **9**, 1–15 (2018).
11. Wu, X. *et al.* Potential for hydrogen-oxidizing chemolithoautotrophic and diazotrophic populations to initiate biofilm formation in oligotrophic, deep terrestrial subsurface waters. *Microbiome* **5**, 1–13 (2017).
12. Daly, R. A. *et al.* Microbial metabolisms in a 2.5-km-deep ecosystem created by hydraulic fracturing in shales. *Nat. Microbiol.* **1**, 16146 (2016).
13. Engelhardt, T., Kallmeyer, J., Cypionka, H. & Engelen, B. High virus-to-cell ratios indicate ongoing production of viruses in deep subsurface sediments. *ISME J.* **8**, 1503–1509 (2014).
14. Borgonie, G. *et al.* Eukaryotic opportunists dominate the deep-subsurface biosphere in South Africa. *Nat. Commun.* **6**, (2015).
15. Jørgensen, B. B. Shrinking majority of the deep biosphere. *Proc. Natl. Acad. Sci. U. S. A.*

109, 15976–15977 (2012).
16. Li, J. *et al.* Recycling and metabolic flexibility dictate life in the lower oceanic crust. *Nature* **579**, 250–255 (2020).
17. Heard, A. W. *et al.* South African crustal fracture fluids preserve paleometeoric water signatures for up to tens of millions of years. *Chem. Geol.* **493**, 379–395 (2018).
18. Robador, A. *et al.* Nanocalorimetric characterization of microbial activity in deep subsurface oceanic crustal fluids. *Front. Microbiol.* **7**, (2016).
19. Bradley, J. A., Amend, J. P. & LaRowe, D. E. Survival of the fewest: Microbial dormancy and maintenance in marine sediments through deep time. *Geobiology* **17**, 43–59 (2019).
20. Wasmund, K., Mußmann, M. & Loy, A. The life sulfuric: microbial ecology of sulfur cycling in marine sediments. *Environ. Microbiol. Rep.* **9**, 323–344 (2017).
21. Momper, L. *et al.* Major phylum-level differences between porefluid and host rock bacterial communities in the terrestrial deep subsurface. *Environ. Microbiol. Rep.* **9**, 501–511 (2017).
22. Suko, T. *et al.* Geomicrobiological properties of Tertiary sedimentary rocks from the deep terrestrial subsurface. *Phys. Chem. Earth* **58**–**60**, 28–33 (2013).
23. Arbour, T. J., Gilbert, B. & Banfield, J. F. Diverse Microorganisms in Sediment and Groundwater Are Implicated in Extracellular Redox Processes Based on Genomic Analysis of Bioanode Communities. *Front. Microbiol.* **11**, (2020).
24. Borgonie, G. *et al.* New ecosystems in the deep subsurface follow the flow of water driven by geological activity. *Sci. Rep.* **9**, 1–16 (2019).
25. Lopez-Fernandez, M., Åström, M., Bertilsson, S. & Dopson, M. Depth and Dissolved Organic Carbon Shape Microbial Communities in Surface Influenced but Not Ancient

Saline Terrestrial Aquifers. *Front. Microbiol.* **9**, 1–16 (2018).

26. Cai, L. *et al.* Active and diverse viruses persist in the deep sub-seafloor sediments over thousands of years. *ISME J.* **13**, 1857–1864 (2019).

27. Lopez-Fernandez, M., Broman, E., Simone, D., Bertilsson, S. & Dopson, M. Statistical analysis of community RNA transcripts between organic carbon and geogas-fed continental deep biosphere groundwaters. *MBio* **10**, e01470-19 (2019).

28. Lopez-Fernandez, M. *et al.* Investigation of viable taxa in the deep terrestrial biosphere suggests high rates of nutrient recycling. *FEMS Microbiol. Ecol.* **94**, 1–9 (2018).

29. Bagnoud, A. *et al.* Reconstructing a hydrogen-driven microbial metabolic network in Opalinus Clay rock. *Nat. Commun.* **7**, 1–10 (2016).

30. Lau, M. C. Y. *et al.* An oligotrophic deep-subsurface community dependent on syntrophy is dominated by sulfur-driven autotrophic denitrifiers. *Proc. Natl. Acad. Sci. U. S. A.* **113**, E7927–E7936 (2016).

31. Lever, M. A. *et al.* Life under extreme energy limitation: A synthesis of laboratory- and field-based investigations. *FEMS Microbiol. Rev.* **39**, 688–728 (2015).

32. Wu, X. *et al.* Microbial metagenomes from three aquifers in the Fennoscandian shield terrestrial deep biosphere reveal metabolic partitioning among populations. *ISME J.* **10**, 1192–1203 (2016).

33. Bell, E. *et al.* Active sulfur cycling in the terrestrial deep subsurface. *ISME J.* **14**, 1260–1272 (2020).

34. Drake, H., Åström, M. E., Tullborg, E. L., Whitehouse, M. & Fallick, A. E. Variability of sulphur isotope ratios in pyrite and dissolved sulphate in granitoid fractures down to 1km depth - Evidence for widespread activity of sulphur reducing bacteria. *Geochim. Cosmochim. Acta* **102**, 143–161 (2013).

35. Alakangas, L. J., Mathurin, F. A. & Åström, M. E. Diverse fractionation patterns of Rare Earth Elements in deep fracture groundwater in the Baltic Shield – Progress from utilisation of Diffusive Gradients in Thin-films (DGT) at the Äspö Hard Rock Laboratory. *Geochim. Cosmochim. Acta* **269**, 15–38 (2020).

36. Drake, H., Tullborg, E. L., Hogmalm, K. J. & Åström, M. E. Trace metal distribution and isotope variations in low-temperature calcite and groundwater in granitoid fractures down to 1km depth. *Geochim. Cosmochim. Acta* **84**, 217–238 (2012).

37. Kärki, A. & Paulamäki, S. *Petrology of Olkiluoto*. *Posiva Report 2006-02. Posiva Oy, Olkiluoto* vol. 2 http://www.posiva.fi/en/databank/posiva_reports/petrology_of_olkiluoto.1871.xhtml?xm_freetext=petrology&xm_col_type=4&cd_order=col_report_number&cd_offset=0#.X44JgS-Q3OQ (2006).

38. Pitkanen, P., Partamies, S. & Luukkonen, A. *Hydrogeochemical Interpretation of Baseline Groundwater Conditions at the Olkiluoto Site. Posiva Report 2003-07, Posiva Oy, Olkiluoto*. http://www.posiva.fi/en/databank/posiva_reports/hydrochemical_interpretation_of_baseline_groundwater_conditions_at_the_olkiluoto_site.1871.xhtml?xm_freetext=Hydrogeochemical+Interpretation+of+Baseline+Groundwater+Conditions+at+the+Olkiluoto+Site&xm_col_ty (2003).

39. Poirier, S. *et al.* Deciphering intra-species bacterial diversity of meat and seafood spoilage microbiota using gyrB amplicon sequencing: A comparative analysis with 16S rDNA V3-V4 amplicon sequencing. *PLoS One* **13**, 1–26 (2018).

40. Caro-Quintero, A. & Ochman, H. Assessing the unseen bacterial diversity in microbial communities. *Genome Biol. Evol.* **7**, 3416–3425 (2015).

41. Li, W. & Godzik, A. Cd-hit: A fast program for clustering and comparing large sets of protein or nucleotide sequences. *Bioinformatics* **22**, 1658–1659 (2006).

42. Hallbeck, L. & Pedersen, K. Characterization of microbial processes in deep aquifers of the Fennoscandian Shield. *Appl. Geochemistry* **23**, 1796–1819 (2008).

43. Drake, H. *et al.* Extreme fractionation and micro-scale variation of sulphur isotopes during bacterial sulphate reduction in deep groundwater systems. *Geochim. Cosmochim. Acta* **161**, 1–18 (2015).

44. Posiva Oy. *Olkiluoto Site Description 2011*. vol. 31 (2011).

45. Laaksoharju, M., Gascoyne, M. & Gurban, I. Understanding groundwater chemistry using mixing models. *Appl. Geochemistry* **23**, 1921–1940 (2008).

46. Mathurin, F. A., Åström, M. E., Laaksoharju, M., Kalinowski, B. E. & Tullborg, E. L. Effect of tunnel excavation on source and mixing of groundwater in a coastal granitoidic fracture network. *Environ. Sci. Technol.* **46**, 12779–12786 (2012).

47. Laaksoharju, M. *et al.* Hydrogeochemical evaluation and modelling performed within the Swedish site investigation programme. *Appl. Geochemistry* **23**, 1761–1795 (2008).

48. Louvat, D., Luc, J. & Franc, J. È spo Origin and residence time of salinity in the A groundwater system. *Appl. Geochemistry* **14**, 917–925 (1999).

49. Li, D., Liu, C.-M. M., Luo, R., Sadakane, K. & Lam, T.-W. W. MEGAHIT: An ultra-fast single-node solution for large and complex metagenomics assembly via succinct de Bruijn graph. *Bioinformatics* **31**, 1674–1676 (2014).

50. Bolger, A. M., Lohse, M. & Usadel, B. Trimmomatic : a flexible trimmer for Illumina sequence data. *Bioinformatics* **30**, 2114–2120 (2014).

51. Kang, D. D., Froula, J., Egan, R. & Wang, Z. MetaBAT, an efficient tool for accurately reconstructing single genomes from complex microbial communities. *PeerJ* **3**, e1165

(2015).

52. Parks, D. H., Imelfort, M., Skennerton, C. T., Hugenholtz, P. & Tyson, G. W. CheckM: assessing the quality of microbial genomes recovered from isolates, single cells, and metagenomes. *Genome Res.* **25**, 1043–55 (2015).

53. Jain, C., Rodriguez-r, L. M. & Aluru, S. High throughput ANI analysis of 90K prokaryotic genomes reveals clear species boundaries. *Nat. Commun.* **9**, 5114 (2018).

54. Parks, D. H. *et al.* A standardized bacterial taxonomy based on genome phylogeny substantially revises the tree of life. *Nat. Biotechnol.* **36**, 996 (2018).

55. Price, M. N., Dehal, P. S. & Arkin, A. P. FastTree 2--approximately maximum-likelihood trees for large alignments. *PLoS One* **5**, e9490 (2010).

56. Hyatt, D. *et al.* Prodigal: prokaryotic gene recognition and translation initiation site identification. *BMC Bioinformatics* **11**, 119 (2010).

57. Huerta-Cepas, J. *et al.* Fast genome-wide functional annotation through orthology assignment by eggNOG-mapper. *Mol. Biol. Evol.* **34**, 2115–2122 (2017).

58. Seemann, T. Prokka: Rapid prokaryotic genome annotation. *Bioinformatics* **30**, 2068–2069 (2014).

59. Lu, S. *et al.* CDD/SPARCLE: The conserved domain database in 2020. *Nucleic Acids Res.* **48**, D265–D268 (2020).

60. Langmead, B. & Salzberg, S. L. Fast gapped-read alignment with Bowtie 2. *Nat. Methods* **9**, 357–359 (2012).

61. Liao, Y., Smyth, G. K. & Shi, W. FeatureCounts: An efficient general purpose program for assigning sequence reads to genomic features. *Bioinformatics* **30**, 923–930 (2014).

62. Rice, P., Longden, I. & Bleasby, A. EMBOSS: The European Molecular Biology Open Software Suite. *Trends Genet.* **16**, 276–277 (2000).

63. Lassmann, T. & Sonnhammer, E. L. L. Kalign--an accurate and fast multiple sequence alignment algorithm. *BMC Bioinformatics* **6**, 298 (2005).

64. Edgar, R. C. MUSCLE: multiple sequence alignment with high accuracy and high throughput. *Nucleic Acid Res.* **32**, 1792–1797 (2004).

65. Kumar, S., Stecher, G. & Tamura, K. MEGA7: Molecular Evolutionary Genetics Analysis Version 7.0 for Bigger Datasets. *Mol. Biol. Evol.* **33**, 1870–1874 (2016).

66. Anantharaman, K. *et al.* Expanded diversity of microbial groups that shape the dissimilatory sulfur cycle. *ISME J.* **12**, 1715–1728 (2018).

67. Nawrocki, E. P. & Eddy, S. R. Infernal 1.1: 100-fold faster RNA homology searches. *Bioinformatics* **29**, 2933–2935 (2013).
