# Supplementary material for: Energy efficiency and biological interactions define the core microbiome of deep oligotrophic groundwater
Source: Nat Commun. 2021 Jul 12;12:4253. doi: 10.1038/s41467-021-24549-z (PMC8275790; doi:10.1038/s41467-021-24549-z)
Supplement: Supplementary file 3 — Description of Additional Supplementary Files [file 41467_2021_24549_MOESM3_ESM.docx]

**Description of Additional Supplementary Files**

Title: Supplementary Data1

Description: Sample, metagenome, metatranscriptome, and Single-cell Amplified genomes information

Title: Supplementary Data2

Description: FSGD MAGs and SAGs stats

Title: Supplementary Data3

Description: SAG clusters

Title: Supplementary Data4

Description: Presence/absence pattern of reconstructed MAGs and SAGs according to mapping results

Title: Supplementary Data5

Description: Origin of available genomes for UBA9089 phylum

Title: Supplementary Data6

Description: KS-D-values

Title: Supplementary Data7

Description: Annotation of transcribed CDSs in Desulfobacterota MAGs andSAG based on HMMER and NCBI conserved domains

Title: Supplementary Data8

Description: Public-goods-annotation identifiers list

Title: Supplementary Data9

Description: Dissimilatory-sulfur-metabolism

Title: Supplementary Data10

Description: Acetate-metabollism-in-patescibacterota

Title: Supplementary Data11

Description: Sporulation-annotation

Title: Supplementary Data12

Description: Toxin-antitoxin-annotation comparison

Title: Supplementary Data13

Description: DnaE2 containing MAGs and SAGs
