# Supplementary material for: Energy efficiency and biological interactions define the core microbiome of deep oligotrophic groundwater
Source: Nat Commun. 2021 Jul 12;12:4253. doi: 10.1038/s41467-021-24549-z (PMC8275790; doi:10.1038/s41467-021-24549-z)
Supplement: Supplementary file 17 — Reporting Summary [file 41467_2021_24549_MOESM17_ESM.pdf]

nature research

Corresponding author(s): Maliheh Mehrshad

Last updated by author(s): Jun 15, 2021

Reporting Summary

Nature Research wishes to improve the reproducibility of the work that we publish. This form provides structure for consistency and transparency in reporting. For further information on Nature Research policies, see our Editorial Policies and the Editorial Policy Checklist.

Statistics

For all statistical analyses, confirm that the following items are present in the figure legend, table legend, main text, or Methods section.

n/a	Confirmed	
☐	☒	The exact sample size (*n*) for each experimental group/condition, given as a discrete number and unit of measurement
☒	☐	A statement on whether measurements were taken from distinct samples or whether the same sample was measured repeatedly
☒	☐	The statistical test(s) used AND whether they are one- or two-sided<br>*Only common tests should be described solely by name; describe more complex techniques in the Methods section.*
☒	☐	A description of all covariates tested
☒	☐	A description of any assumptions or corrections, such as tests of normality and adjustment for multiple comparisons
☒	☐	A full description of the statistical parameters including central tendency (e.g. means) or other basic estimates (e.g. regression coefficient) AND variation (e.g. standard deviation) or associated estimates of uncertainty (e.g. confidence intervals)
☒	☐	For null hypothesis testing, the test statistic (e.g. *F*, *t*, *r*) with confidence intervals, effect sizes, degrees of freedom and *P* value noted<br>*Give P values as exact values whenever suitable.*
☒	☐	For Bayesian analysis, information on the choice of priors and Markov chain Monte Carlo settings
☒	☐	For hierarchical and complex designs, identification of the appropriate level for tests and full reporting of outcomes
☒	☐	Estimates of effect sizes (e.g. Cohen's *d*, Pearson's *r*), indicating how they were calculated

Our web collection on statistics for biologists contains articles on many of the points above.

Software and code

Policy information about availability of computer code

Data collection	No software was used for data collection.
Data analysis	We have used the following programs to analyze the data in this study.<br>MEGAHIT (v. 1.1.3)<br>MetaBAT2 (v. 2.12.1)<br>CheckM (v. 1.0.7)<br>GTDB-tk (v. 0.2.2)<br>FastTree (v. 2.1.10)<br>Trimmomatic (v. 0.36)<br>Prodigal (v. 2.6.2)<br>eggnog-mapper (v. 2.2.1)<br>pfam_scan.pl (v. 1.6)<br>Prokka (v. 1.12)<br>bowtie2 (v. 2.3.3.1)<br>MarkDuplicates from the picard suite (v. 2.18.6)<br>featureCounts (v. 1.6.1)<br>pepstat and cusp software in the EMBOSS package (v. 6.6.0)<br>cmsearch (v. 1.1.3)<br>Kalign (v. 2.04)<br>fastANI (v. 1.1)<br>nucmer (v. 3.23)<br>MEGA 7

we used these data sources:
eggnog_5.0 database (http://eggnog5.embl.de/#/app/home)
31.0 release of the PFAM database (http://ftp.ebi.ac.uk/pub/databases/Pfam/releases/Pfam31.0/)
the thresholds for each analysis are mentioned in the text and supplementary information in detail. No custom code that is central to the analyses of this study has been generated.

For manuscripts utilizing custom algorithms or software that are central to the research but not yet described in published literature, software must be made available to editors and reviewers. We strongly encourage code deposition in a community repository (e.g. GitHub). See the Nature Research guidelines for submitting code & software for further information.

Data

Policy information about availability of data

All manuscripts must include a data availability statement. This statement should provide the following information, where applicable:
- Accession codes, unique identifiers, or web links for publicly available datasets
- A list of figures that have associated raw data
- A description of any restrictions on data availability

Metagenomes, SAGs, MAGs, and metatranscriptomes that support the findings of this study are deposited in GenBank and their respective accession numbers are provided in the Supplementary Data 1. The FSGD MAGs are deposited in GenBank under the NCBI BioProject with the accession number PRJNA627556 (https://www.ncbi.nlm.nih.gov/bioproject/PRJNA627556). The MAGs and SAGs generated in this study are publicly available in figshare under the project “Fennoscandian Shield genomic database (FSGD)” with the identifier DOI:10.6084/m9.figshare.12170313. Alignments and phylogenetic trees that support the findings of this study are available in figshare under the project “Fennoscandian Shield genomic database (FSGD)” with the identifiers DOI:10.6084/m9.figshare.14166650, DOI:10.6084/m9.figshare.14166638, DOI:10.6084/m9.figshare.13298513, and DOI:10.6084/m9.figshare.12170310. All data supporting the findings of this paper are available within this paper and its supplementary material. All the programs used and the version and set thresholds are mentioned in the manuscript, supplementary information, and the reporting summary. Source data are provided with this paper.

Field-specific reporting

Please select the one below that is the best fit for your research. If you are not sure, read the appropriate sections before making your selection.

☐ Life sciences ☐ Behavioural & social sciences ☒ Ecological, evolutionary & environmental sciences

For a reference copy of the document with all sections, see nature.com/documents/nr-reporting-summary-flat.pdf

Ecological, evolutionary & environmental sciences study design

All studies must disclose on these points even when the disclosure is negative.

Study description

This study is an exploratory research to investigate the existence of a common microbiome in the deep groundwaters running through a similar lithology. This study does not include treatment factors and interactions, design structure (factorial, nested, heirarchical). The metatranscriptome samples were collected in replicates and detailed information is mentioned in the text and supplementary information.

Research sample

Multiple groundwater samples were collected over several years from two deep geological sites excavated in crystalline bedrock of the Fennoscandian Shield. These sites provide access to deep groundwater flowing in similar lithology. in The first is the Swedish Nuclear Fuel and Waste Management Company (SKB) operated Äspö HRL located in the southeast of Sweden (Lat N 57° 26’ 4’’ Lon E 16° 39’ 36’’). The second site is on the island of Olkiluoto, Finland, that will also host a deep geological repository for the final disposal of spent nuclear fuel (Lat N 61° 14’ 31’’, Lon E 21° 29’ 23’’). There are limited number of access points to study the deep groundwater microbime these two sites provide access to groundwater flowing in similar lithologies. These samples allow for exploring the microbial diversity of deep disconnected groundwaters and understanding whether they harbor a common core microbiome. The collected groundwater samples were used for metagenomics sequencing (n=27 from the Äspö HRL and n=17 from Olkiluoto), single cell genomics (n=564), and metatranscriptomics (n=9 from the Äspö HRL). All samples are deposited to the National Center for Biotechnology Information (NCBI). Detailed information and accession numbers are provided in the text and Supplementary Data1.

Sampling strategy

Samples of groundwater flowing in bedrock fractures of the Fennoscandian Shield were collected from two sites with similar lithologies. The first is the Swedish Nuclear Fuel and Waste Management Company (SKB) operated Äspö HRL located in the southeast of Sweden (Lat N 57° 26’ 4’’ Lon E 16° 39’ 36’’). The second site is on the island of Olkiluoto, Finland, that will also host a deep geological repository for the final disposal of spent nuclear fuel (Lat N 61° 14’ 31’’, Lon E 21° 29’ 23’’). We used available metatranscriptomes collected from the Äspö HRL to check for the active metabolism. No statistical method was used to determine the sample size. In order to make sure we will be able to extract enough DNA/RNA for sequencing from the collected samples, we continued filtration until the filters were clogged. We sampled each borehole more than once to study their composition at different time points.

Data collection

Water samples for generating metagenomes were collected from the Äspö Hard Rock Laboratory (Äspö HRL) from boreholes SA1229A-1 (171.3 mbsl), KA3105A-4 (415.2 mbsl), KA2198A (294.1 mbsl), KA3385A-1 (448.4 mbsl), and KF0069A01 (454.8 mbsl). Planktonic cells were collected after flushing five borehole section volumes on sterile polyvinylidene fluoride (PVDF), hydrophilic, 0.1 µm, 47 mm Durapore membrane filters (Merck Millipore) under in situ conditions by connecting a High-Pressure Stainless Steel Filter Holder (Millipore) with a downstream needle valve and pressure gauge directly to the borehole. After filtering an appropriate volume of groundwater, each filter was rolled and placed in a sterile cryogenic tube (Thermo Scientific) and immediately frozen in liquid nitrogen. Samples were frozen at the sampling site to allow transport to the laboratory without any changes in the microbial community. Tubes were stored at -80 °C until further processing.

Samples were collected and processed by the Mark Dopson lab at the Linnaeus University.

The island of Olkiluoto on the south-west coast of Finland groundwater was collected from three drillholes that access fracture fluids at different depths; OL-KR11 (366.7-383.5 mbsl), OL-KR13 (330.5-337.9 mbsl), and OL-KR46 (528.7-531.5 mbsl). Multiple samples were collected during 2016 (OL-KR11 n = 7, OL-KR13 n = 7 and OL-KR46 n = 3). To collect biomass for DNA analysis, approximately 10 L of groundwater was pumped directly into a chilled sterile Nalgene filtration unit fitted with a 0.22 µm pore size Isopore polycarbonate membrane (Millipore) and connected to a vacuum pump. After filtration, the membrane filters were rolled and stored in 1.5 mL sterile screwcap tubes. Filters collected for DNA extraction were preserved in 750 mL LifeGuard Soil Preservation Solution (MoBio, Carlsbad, CA, United States) and transferred to the laboratory on dry ice. Filters were stored at -20 °C until further processing.
Samples were collected and processed by the Rizlan Bernier Latmani Lab at the École Polytechnique Fédérale de Lausanne.

Water samples for capturing single-cell amplified genomes (SAGs) were collected from KA3105A-4 (n=15), KA3385A-1 (n=148), SA1229A-1 (n=118), OL-KR11 (n=138), OL-KR13 (n=117), and OL-KR46 (n=28) water samples. SAGs were amplified, sequenced, and assembled by the Joint Genome Institute (JGI), USA.
samples were collected and processed according to the JGI guidelines by Margarita Lopez-Fernandez, Mark Dopson, and Emma Bell.

Metatranscriptomic datasets were generated for Äspö HRL water samples originating from boreholes KA3105A-4 (n=2), KA3385A-1 (n=4), and SA1229A-1 (n=3). The groundwaters were sampled from the Äspö HRL under in situ conditions using two different sampling methods. Firstly, by connecting a RNA sampling device with an in-built fixation system (constructed by Maskinteknik AB, Oskarshamn, Sweden) from June 2015 to March 2016. Secondly, by connecting a high-pressure stainless steel filter holder (Merck Millipore, USA) with a downstream needle valve and pressure gauge from September 2015 to January 2016. detailed information and appropriate references are mentioned in the supplementary information and Supplementary Data1. In both cases, planktonic cells were collected on sterile hydrophilic polyvinylidene fluoride (PVDF) membranes with 0.1 µm poresize (47 mm Durapore, Merck Millipore, USA) under in situ conditions.
Samples were collected and processed by the Mark Dopson lab at the Linnaeus University.

Timing and spatial scale	Metagenomics amples were collected from the Äspö Hard Rock Laboratory (Äspö HRL) during 2013, 2014 and 2016. The Olkiluoto samples were collected during 2016. Metatranscriptomic datasets were generated during the 2015 and 2016. This study was designed to explore the diversity of the deep groundwater samples in different aquifers.
Data exclusions	No data were excluded from the analyses.
Reproducibility	Sample collection and processing of the two locations has been done independently and by two different groups. Analyses of this study are performed independently for each dataset. All methods used in this study are referenced extensively and results have been reproduced by recovering similar metagenome assembled genomes from different sites. All the data are publicly available and all methods with the parameters used are mentioned in the manuscript and supplemental information. The raw data for figures are submitted as Source Data and Supplementary Data.
Randomization	Randomization is not relevant to the nature of this study. In this study we explore the microbial community of the deep groundwater in two disconnected locations. samples originating from each location are analyzed independently and results are compared to find overlaps that is defined as the common core microbiome between the two locations.
Blinding	This study is an exploratory study analyzing the microbial diversity and there was no prior expectation on what would influence the results, hence no blinding was performed in this study.

Did the study involve field work? ☒ Yes ☐ No

Field work, collection and transport

Field conditions	Multiple groundwater samples were collected over several years from two deep geological sites excavated in crystalline bedrock of the Fennoscandian Shield. The first is the Swedish Nuclear Fuel and Waste Management Company (SKB) operated Äspö HRL located in the southeast of Sweden (Lat N 57° 26′ 4′′ Lon E 16° 39′ 36′′). The second site, operated by Posiva Oy, is on the island of Olkiluoto, Finland and will also host a deep geological repository for the final disposal of spent nuclear fuel (Lat N 61° 14′ 31′′, Lon E 21° 29′ 23′′). Water types with various ages and origins were targeted by sampling fracture fluids from different depths. detailed information are available in Supplementary methods and Supplementary Table S1.
Location	Metagenomics amples were collected from the Äspö Hard Rock Laboratory (Äspö HRL). This underground laboratory located in the south-east of Sweden (Lat N 57° 26′ 4″ Lon E 16° 39′ 36″) is excavated in the Proterozoic crystalline bedrock of the Fennoscandian Shield extending to a depth of 460 m below sea level (mbsl) with 3600 m of total tunnel length. The Äspö HRL samples originated from boreholes SA1229A-1 (171.3 mbsl), KA3105A-4 (415.2 mbsl), KA2198A (294.1 mbsl), KA3385A-1 (448.4 mbsl), and KF0069A01 (454.8 mbsl).<br>The island of Olkiluoto on the south-west coast of Finland will host a deep geological repository for the final disposal of spent nuclear fuel (Lat N 61° 14′ 31′′, Lon E 21° 29′ 23′′). Groundwater was collected from three drillholes that access fracture fluids at different depths; OL-KR11 (366.7-383.5 mbsl), OL-KR13 (330.5-337.9 mbsl), and OL-KR46 (528.7-531.5 mbsl). Multiple samples were collected during 2016 (OL-KR11 n = 7, OL-KR13 n = 7 and OL-KR46 n = 3).<br><br>Single-cell amplified genomes (SAGs) were captured from KA3105A-4 (n=15), KA3385A-1 (n=148), SA1229A-1 (n=118), OL-KR11 (n=138), OL-KR13 (n=117), and OL-KR46 (n=28) water samples.<br><br>Metatranscriptomic datasets were generated for Äspö HRL samples originating from boreholes KA3105A-4 (n=2), KA3385A-1 (n=4), and SA1229A-1 (n=3).

	Details of sampling, filtration, DNA/RNA processing, and geochemical parameters of the water samples along with statistics of the metagenomics/metatranscriptomics datasets and SAGs are available in Supplementary methods and Supplementary Data1.
Access & import/export	The study was covered by activity plans for work in the Äspö Hard Rock Laboratory. Samples were filtered and processed by the Mark Dopson lab. Olkiluoto samples were collected in collaboration with Posiva Oy, filtered and processed by the Rizlan Bernier-Latmani Lab.
Disturbance	No disturbances were caused by the sampling.

Reporting for specific materials, systems and methods

We require information from authors about some types of materials, experimental systems and methods used in many studies. Here, indicate whether each material, system or method listed is relevant to your study. If you are not sure if a list item applies to your research, read the appropriate section before selecting a response.

Materials & experimental systems

n/a	Involved in the study	
☒	☐	Antibodies
☒	☐	Eukaryotic cell lines
☒	☐	Palaeontology and archaeology
☒	☐	Animals and other organisms
☒	☐	Human research participants
☒	☐	Clinical data
☒	☐	Dual use research of concern

Methods

n/a	Involved in the study	
☒	☐	ChIP-seq
☒	☐	Flow cytometry
☒	☐	MRI-based neuroimaging
